# Effects of atomoxetine and methylphenidate on quality of life and self-esteem in adults with ADHD

**DOI:** 10.3389/fpsyt.2026.1866964

**Published:** 2026-08-07

**Authors:** Rosaria Di Lorenzo, Jessica Bonisoli, Federica Gualtieri, Emanuela Latella, Isabella Riguzzi, Paola Ferri, Sergio Rovesti

**Affiliations:** 1 Department of Mental Health and Drug Abuse, AUSL-Modena, Modena, Italy; 2 School of Specialization in Psychiatry, University of Modena and Reggio Emilia, Reggio Emilia, Italy; 3 Department of Biomedical, Metabolic and Neural Sciences, University of Modena and Reggio Emilia, Modena, Italy

**Keywords:** adult ADHD, atomoxetine, methylphenidate, quality of life, self-esteem

## Abstract

**Objective:**

Attention-Deficit/Hyperactivity Disorder (ADHD) in adulthood is a complex neuropsychiatric condition characterized by inattention, hyperactivity/impulsivity, often associated with emotional dysregulation, with a significant impact on global functioning, self-esteem, and quality of life. The aim of this study is to compare the effects of atomoxetine and methylphenidate on quality of life and self-esteem in adults diagnosed with ADHD.

**Methods:**

A naturalistic observational study was conducted on a sample of adults, divided into methylphenidate and atomoxetine treatment groups. Assessments were performed at baseline (T0) and after a mean period (T1) of 176.37 (± 173.83 SD) days using Barkley Adult ADHD Rating Scale-IV (BAARS-IV) Quick Screen, Adult ADHD Quality of Life (AAQoL-29), Rosenberg Self-Esteem Scale (RSES), and Global Assessment Functioning (GAF).

**Results:**

At T1, we showed a significant improvement in both groups in all dimensions: reduction of ADHD symptoms (BAARS total: p = 0.012), increase in overall quality of life (AAQoL-29 total: p < 0.001) and global functioning (GAF: p < 0.001), as well as an increase in self-esteem scores (RSES total: p = 0.0002). Methylphenidate proved more effective than atomoxetine in improving both quality of life (total AAQoL-29: p = 0.006) and self-esteem (total RSES: p = 0.031). Correlation analysis showed that symptom severity, assessed with the DIVA 5 test, was inversely proportional to levels of self-esteem (Spearman rho = -0.3269; p = 0.0131) and quality of life (Spearman rho = -0.3758; p = 0.0040) and that RSES and AAQoL-29 scores were positively correlated (Spearman rho = 0.6357; p = 0.0000).

**Conclusion:**

Our study confirms that pharmacological therapy in adult ADHD not only improves core symptoms but also promotes improvement in self-esteem and personal efficacy, essential components for the recovery of psychological and social well-being.

## Introduction

Attention-Deficit/Hyperactivity Disorder (ADHD) is a neurodevelopmental disorder characterized by a persistent pattern of inattention, hyperactivity, and impulsivity, with typical onset in childhood and possible persistence into adulthood (1, 2). Although initially considered a predominantly paediatric condition, substantial epidemiological and clinical evidence has demonstrated that a significant proportion of individuals continue to exhibit clinically relevant symptoms in adulthood, with persistence rates estimated between 30% and 85% depending on diagnostic criteria and study contexts (3–8).

In adults, the clinical presentation of ADHD differs from that observed in childhood, being less dominated by motor hyperactivity and more characterized by inattention, organizational difficulties, inner restlessness, and emotional dysregulation (6, 9, 10). These symptoms are frequently associated with deficits in executive functions, including reduced inhibitory control, working memory impairment, and planning difficulties, which substantially contribute to global functional impairment (11–14).

Adult ADHD is associated with a significant impact on occupational, social, and relational functioning, with high rates of job instability, interpersonal conflict, and risk-taking behaviour (9, 12). These difficulties translate into a marked reduction in Quality of Life (QoL), according to the definition of World Health Organization (15), making this construct a clinically relevant outcome.

Numerous studies have shown that individuals with ADHD across all age groups report significantly lower QoL compared to the general population, particularly in the domains of productivity, interpersonal relationships, and emotional well-being (6, 16, 17). Alongside QoL, self-esteem represents a central psychological dimension in ADHD. Repeated experiences of academic and occupational failure, negative feedback, and difficulties in social adaptation contribute to the development of a fragile self-image and persistent feelings of inadequacy (18, 19).

The literature suggests that impairment in quality of life and self-esteem among individuals with ADHD cannot be explained solely by the severity of core symptoms but is also mediated by executive function deficits and emotional dysregulation (16, 20, 21). These factors hinder self-regulation and the ability to manage daily demands, negatively affecting subjective well-being even in the presence of partial symptom reduction.

Pharmacological treatment represents one of the cornerstones of ADHD management. From a neurobiological perspective, the disorder is associated with dysregulation of dopaminergic and noradrenergic systems in the prefrontal cortex, a region crucial for executive functioning and self-regulation (22, 23).

Methylphenidate, the primary stimulant medication used in ADHD treatment, acts by inhibiting dopamine and norepinephrine reuptake, increasing the synaptic availability of these neurotransmitters and improving the efficiency of frontal-striatal circuits (24). Numerous randomized controlled trials and meta-analyses have confirmed its efficacy in reducing core ADHD symptoms (25, 26).

Atomoxetine, a selective norepinephrine reuptake inhibitor, represents an important therapeutic alternative, particularly in adulthood and in individuals with contraindications to stimulants. Its mechanism of action increases norepinephrine and dopamine levels in the prefrontal cortex without activating mesolimbic dopaminergic circuits, thereby reducing abuse potential (27, 28). The pharmacological profile of atomoxetine has been associated with gradual but stable improvements in emotional regulation and overall functioning (9).

Although the efficacy of methylphenidate and atomoxetine in reducing ADHD symptomatology is well established, their impact on subjective outcomes such as quality of life and self-esteem remains less clearly defined, particularly in adults. Some evidence suggests that stimulants may produce more rapid effects on productivity and daily functioning, whereas non-stimulants may promote more progressive and sustained emotional benefits (16, 25). However, direct comparative data remain limited.

In light of these considerations, it is essential to further investigate the effects of pharmacological treatment with methylphenidate and atomoxetine on quality of life and self-esteem in adults with ADHD, in order to guide increasingly personalized therapeutic choices focused not only on symptom control but also on improving psychological well-being and overall functioning.

## Methods

### Study design and clinical setting

An observational, longitudinal, retrospective–prospective, cohort, single-center, non-profit study was conducted to evaluate the effects of pharmacological treatment with methylphenidate and atomoxetine on quality of life, self-esteem, and global functioning in adult patients with Attention-Deficit/Hyperactivity Disorder (ADHD).

The study was carried out at the ADHD Adult Center (ADHD-AC) of the Department of Mental Health and Drug Abuse of AUSL-Modena.

The study protocol was implemented over a total period of 20 months, from March 2024 to October 2025, including sample recruitment, baseline clinical assessment, follow-up, statistical analysis, and data processing. Patients who discontinued pharmacological treatment (drop-outs) were included in the analyses to provide a representation more consistent with real-world clinical practice.

### Sample selection

Patients of both sexes referred to the ADHD-AC were enrolled. Inclusion criteria were: age ≥18 years; residence in Modena and its province; diagnosis of adult ADHD established through structured clinical evaluation and validated psycho-diagnostic instruments; provision of informed consent for participation and data processing.

Patients were referred to the ADHD-AC by general practitioners, community mental health services, or private practice specialists. Individuals were excluded if they did not meet diagnostic criteria for adult ADHD, did not initiate specific pharmacological treatment (reserved for patients with moderate-to-severe functional impairment in at least two life domains), or presented language barriers or moderate-to-severe cognitive impairment. During the prospective recruitment period, 167 individuals accessed the ADHD-AC. Among those for whom pharmacological treatment was indicated, 50.1% were treated with atomoxetine and 49.9% with methylphenidate. The choice of which drug to prescribe was determined by the following criteria: metylphenidate was considered the first-choice drug conditioned by its availability (in Italy, methylphenidate was authorized for adult ADHD in October 2023) and substance use or toxicophilic attitude of the patient (in these cases only atomoxetine was prescribed).

### Assessment instruments

Initial screening for adult ADHD was performed using the Adult ADHD Self-Report Scale version 1.1 (ASRS v1.1), a self-administered questionnaire consisting of 18 items corresponding to DSM-IV-TR diagnostic criteria (29), with a 6-item short form (Part A) demonstrating high predictive validity (30, 31). The ASRS v1.1 was used as a first-level screening tool to identify individuals at high risk for ADHD, consistent with clinical practice and international recommendations.

In case of positive screening, assessment was further refined using the Barkley Adult ADHD Rating Scale-IV (BAARS-IV) Self-Report (32). The BAARS-IV provides a detailed self-evaluation of ADHD symptoms in both adulthood and childhood, exploring symptom frequency, age at onset, and functional impact across life domains. A distinctive feature of this instrument is the inclusion of a section dedicated to Sluggish Cognitive Tempo (SCT) (33), a construct frequently associated with ADHD and relevant for the clinical characterization of adult patients with ADHD.

The diagnosis was ultimately confirmed using the Diagnostic Interview for ADHD in Adults (DIVA-5) (34), a semi-structured clinical interview based on DSM-5 criteria, validated in Italian (35). The DIVA-5 allowed us systematic assessment of inattention and hyperactivity/impulsivity symptoms in adulthood and during childhood (ages 5-12), integrating concrete examples across major functional domains (work, relationships, daily life). This instrument was employed to ensure high diagnostic accuracy and standardization of the assessment procedure.

At follow-up (T1), ADHD symptomatology was reassessed using the BAARS-IV Self-Report (32): Quick Screen, a brief 6-item self-administered version that enables rapid and reliable evaluation of core ADHD symptoms and is particularly suitable for clinical follow-up contexts.

QoL was assessed using the Adult ADHD Quality of Life scale (AAQoL-29), a self-administered questionnaire specifically developed for adults with ADHD (36). The AAQoL-29 consists of 29 items divided into four domains: Life Productivity, Psychological Health, Relationships, and Life Outlook. Each item is rated on a 5-point Likert scale, yielding domain scores and a total score, with higher values indicating better perceived quality of life. The score was successively converted from 1-to-5 numbers to a new 0-to-100 scale. This instrument was selected for its ADHD-specific focus and strong psychometric properties highlighted by international validation studies (37, 38).

Self-esteem was measured using the Rosenberg Self-Esteem Scale (RSES) (39), one of the most widely used instruments worldwide for assessing global self-esteem (40). The scale consists of 10 items rated on a 4-point Likert scale (Strongly Agree = 0 points, Agree = 1 point, Disagree = 2 points, Strongly Disagree = 3 points, final scoring 0-30) and provides a total score as well as subcomponents related to personal competence and self-satisfaction. The RSES was selected for its brevity, reliability, cross-population validity, and availability of normative data for adult populations (41).

Global functioning was assessed by clinicians using the Global Assessment of Functioning (GAF) (29), a scale providing a single summary measure of psychological, social, and occupational functioning on a continuum from 1 to 100. The GAF integrates symptom severity and functional level into a single score, making it useful for monitoring clinical progress over time.

### Selected variables

Variables included sociodemographic (age, sex, nationality, living arrangement, marital status, educational level, employment status) and clinical (personal and familial anamnestic information, pharmacological, educational and rehabilitative treatments, psychiatric hospitalizations, childhood clinical care, age at first clinical care, psychiatric comorbidities) data.

Treatment drop-outs due to adverse events, lack of efficacy, or poor adherence were also recorded.

### Assessment procedures

Data collection was conducted at two time points:

Baseline assessment (T0): At intake, anamnestic, sociodemographic, and clinical data were collected through a semi-structured interview. Psycho-diagnostic questionnaires and outcome scales (ASRS v1.1, DIVA-5, BAARS-IV, AAQoL-29, RSES, and GAF) were administered at this stage.

Follow-up (T1): At the end of the clinical observation period, patients were reassessed using self-report instruments evaluating ADHD symptomatology (BAARS-IV Self-Report: Quick Screen), quality of life (AAQoL-29), and self-esteem (RSES); global functioning (GAF) was reassessed by the clinician.

Data comparing sex difference and the two treatments (methylphenidate vs atomoxetine) and difference between T0 and T1 were analyzed.

### Statistical analysis

Data were anonymized and entered an electronic database. Descriptive analyses of sample characteristics were conducted. Continuous variables were described using means and standard deviations and compared using t-tests; categorical variables were analyzed using percentage frequencies and Pearson’s chi-square tests. Correlations between ADHD symptomatology, quality of life, self-esteem, and global functioning were explored using Spearman’s correlation coefficient. Normality of distributions was assessed using the Shapiro–Wilk test. Statistical significance was set at p < 0.05. Analyses were performed using STATA software, version 19 (2025).

### Ethical considerations

The study was conducted in accordance with the Declaration of Helsinki and was approved by the competent Ethics Committee (Comitato Etico Area Vasta Emilia Nord: Prot. AOU 0010629/25, 09/04/2025) and authorized by AUSL-Modena (n. 1702, 26-6-2024). All participants provided written informed consent. Data processing was carried out in compliance with European Regulation 679/2016 (GDPR) and current Italian data protection legislation.

## Results

### Socio-demographic characteristics of the sample

The study sample consisted of 57 adult patients diagnosed with Attention-Deficit/Hyperactivity Disorder (ADHD), including 27 males (47.4%) and 30 females (52.6%), as reported in **Table 1**. The overall mean age was 29.26 years (SD = 7.9), with slightly lower mean values in males than females, although the difference was not statistically significant. Most participants were Italian nationals (87.7%), while a minority were of non-Italian Europeans (3.5%) or non-European origin (8.8%). Regarding marital status, single individuals predominated (59.6%), followed by married or cohabiting participants (38.6%); only one patient was divorced or separated. Educational attainment was generally high: 47.4% had completed high school and 19.3% held a university degree. In terms of employment status, students represented the largest group (43.9%), followed by employees (36.8%), unemployed individuals (14.0%), and self-employed professionals (5.3%). No participants were retired. Regarding living arrangements, approximately half of the sample (49.1%) lived with their family of origin, while the remainder lived with their own family (29.8%) or independently (21.1%). Comparative analyses revealed no statistically significant gender differences across socio-demographic variables, confirming sample homogeneity.

**Table 1:** Socio-demographic variables of the sample stratified by sex.

	Males (n= 27)	Females (n=30)	Total (n= 57)	Statistical test	Probability
Nationality, n (%)					
Italian	24 (88.9%)	26 (86.7%)	50 (87.7%)	Pearson chi2 = 0.12	p = 0.941
European	1 (3.7%)	1 (3.3%)	2 (3.5%)		
Non-European	2 (7.4%)	3 (10%)	5 (8.8%)		
Marital Status, n (%)					
Single	17 (63%)	17 (56.7%)	34 (59.6%)	Pearson chi2 = 1.57	p = 0.455
Married/cohabiting	9 (33.3%)	13 (43.3%)	22 (38.6%)		
Divorced/separated	1 (3.7%)	0 (0%)	1 (1.8%)		
Education level, n (%)					
Primary school	0 (0%)	0 (0%)	0 (0%)	Pearson chi2 = 0.75	p = 0.687
Middle school	10 (37%)	9 (30%)	19 (33.3%)		
High school	13 (48.1%)	14 (46.7%)	27 (47.4%)		
University degree	4 (14.8%)	7 (23.3%)	11 (19.3%)		
Employment status, n (%)					
Employed	9 (33.3%)	12 (40%)	21 (36.8%)	Pearson chi2 = 6.61	p = 0.085
Independent contractor	2 (7.4%)	1 (3.3%)	3 (5.3%)		
Unemployed	4 (14.8%)	4 (13.3%)	8 (14.0%)		
Retired	0 (0%)	0 (0%)	0 (0%)		
Student	12 (44.4%)	13 (43.3%)	25 (43.9%)		
Living arrangement, n (%)					
Living alone	6 (22.2%)	6 (20%)	12 (21.1%)	Pearson chi2 = 2.32	p = 0.313
With family of origin	15 (55.6%)	13 (43.3%)	28 (49.1%)		
With own family	6 (22.2%)	11 (36.7%)	17 (29.8%)		
Age, years (mean ± SD)	27.81 ± 7.88	30.57 ± 7.89	29.26 ± 7.9	*t* = 1.56. *t-*test	p = 0.456

The comparison between the two treatment groups, methylphenidate vs atomoxetine, shows that they were composed of 28 and 29 individuals respectively: more males (n=17, 65.38%) were treated with methylphenidate whereas more females (n=20, 64.52%) were treated with atomoxetine (Pearson Chi2 = 3.93; p = 0.047). The socio-demographic variables were similar between the two treatment groups, being statistically significantly different only among the employment status: more independent contractors were present in the methylphenidate group (Pearson Chi2 = 10.01, p = 0.018).

### Clinical variables

Our sample consisted of 57 individuals: 35 of them were diagnosed with ADHD inattentive (13 males and 22 females) and 22 with ADHD combined (14 males and 8 females), with a statistically significant difference between the two sexes (Pearson chi2 = 3.803, p = 0.050) but not between the two treatment groups, as reported in **Table 2**.

**Table 2:** Clinical variables of the sample divided into the two treatment groups at T0.

	Methylphenidate (n= 28)	Atomoxetine (n=29)		Total (n= 57)	Statistical test	Probability
ADHD types, n (%)						
Inattentive (n = 35)	18 (64.29%)	17 (58.62%)		35 (61.4%)	Pearson chi2 = 0.19	p = 0.661
Combined (n= 22)	10 (35.71%)	12 (41.38%)		22 (38.6%)		
Associated psychiatric diagnoses, n (%)						
None	13 (46.13%)	8 (27.59%)		20 (36.84%)	Pearson chi2= 7.1611	p = 0.209
Depression / Anxiety	10 (35.71%)	9 (31.03%)		19 (33.33%)		
Personality disorder	3 (10.71%)	7 (24.14%)		10 (17.54%)		
Bipolar disorder	0 (0%)	3 (10.34%)		3 (5.26%)		
Autism spectrum disorder	2 (7.14%)	1 (3.45%)		3 (5.26%)		
Obsessive-compulsive disorder (OCD)	0 (0%)	1 (3.45%)		1 (1.75%)		
Current pharmacological treatment, n (%)						
None	18 (69.70%)*	5 (17.24%)		23 (40.35%)	Pearson chi2 = 57.00 *RS≥2, p<0.05	p < 0.001
Antidepressants	9 (27.27%)*	0 (0%)		9 (15.79%)		
Antipsychotics	0 (0%)	24 (82.76%)*		24 (42.11%)		
Polypharmacotherapy	1 (3.03%)	0 (0%)		1 (1.75%)		
Substance abuse, n (%)						
None	27 (96.43%)	25 (86.21%)		52 (91.23%)	Pearson chi2 = 2.39	p = 0.495
THC	1 (3.57%)	2 (6.90%)		3 (5.26%)		
Alcohol	0 (0%)	1 (3.45%)		1 (1.75%)		
Psychotropic medications	0 (0%)	1 (3.45%)		1 (1.75%)		
Medical comorbidities, n (%)						
Present	1 (3.57%)	2 (6.90%)		3 (5.26%)	Pearson chi2 = 0.32	p = 0.574
Not present	27 (96.43%)	27 (93.10%)		54 (84.74%)		
Outpatient service treatment, n (%)						
Present	13 (46.43%)	21 (72.41%)		34 (59.56%)	Pearson chi2 = 3.10	p = 0.046
Not Present	15 (53.57%)	8 (27.59%)		23 (40.35%)		
Previous psychiatric hospitalization, n (%)						
Present	2 (7.14%)	7 (24.14%)		9 (15.79%)	Pearson chi2 = 3.09	p = 0.079
Not Present	26 (92.86%)	22 (75.86%)		48 (84.21%)		

From an anamnestic perspective, more than one-third of participants (35.09%) reported a family history of psychiatric disorders, as shown in **Table 3**. Adverse childhood experiences were present in 52.63% of cases, mainly parental separation (17.54%). Less than half of the sample (49.12%) had participated in educational or rehabilitative programs during childhood, with a significant statistical difference between the two treatment groups (75% of atomoxetine group vs 25% of methylphenidate group, Pearson chi2 = 12.81, p = 0.000), as reported in **Table 3**.

**Table 3:** Childhood/adolescence variables of the sample divided into the two treatment groups.

		Methylphenidate (n= 28)	Atomoxetine (n=29)	Total (n= 57)	Statistical test	Probability
Family history, n (%)						
None		15 (45.45%)	18 (54.55%)	33 (58%)	Pearsonchi2=2.39	p = 0.496
Psychiatric disorders		12 (60%)	8 (40%)	20 (35%)		
Substance use		1 (3.33%)	2 (6.67%)	3 (5%)		
Both psychiatric and substance use disorders		0 (0%)	1 (100%)	1 (2%)		
Other medical conditions		0 (0%)	0 (0%)	0 (0%)		
Adverse childhood experiences, n (%)						
Adoption		1 (50%)	1 (50%)	2 (3.51%)	Pearsonchi2=8.20	p = 0.224
Bereavement of a family member		0 (0%)	3 (100%)	3 (5.26%)		
Parental separation		4 (40%)	6 (60%)	10 (17.54%)		
Physical illness		0 (0%)	2 (100%)	2 (3.51%)		
Socio-economic difficulties		0 (0%)	1 (100%)	1 (1.75%)		
Other		6 (50%)	6 (50%)	12 (21.05%)		
None		17 (62.96%)	10 (37.04%)	27 (47.37%)		
Educational/rehabilitative programs, n (%)						
Yes		7 (25%)	21 (75%)	28 (49.12%)	Pearsonchi2=12.81	p < 0.001
No		21 (72.41%)	8 (27.59%)	29 (50.88%)		
Childhood clinical care, n (%)						
Yes		13 (59.09%)	9 (40.91%)	22 (38.60%)	Pearsonchi2=1.42	p = 0.233
No		15 (42.86%)	20 (57.14%)	35 (61.40%)		
Age at first clinical care, years (mean ± SD)						
		12.23 ± 4.62	6.59 ± 1.30	8.33	*t* = 6.14, *t*-test	p < 0.001
Childhood community service treatment, n (%)						
Present		13 (46.43%)	9 (31.03%)	22 (38.60%)	Pearson chi2 = 1.42	p = 0.233
Not present		15 (53.57%)	20 (68.97%)	35 (61.40%)		

The mean age at first intervention was 12.45 ± 4.20 years, with a significant statistical difference between the two treatment groups (6.59 ± 1.30 years in atomoxetine group vs 12.23 ± 4.62 in methylphenidate group, t = 6.14, t-test, p <0.001), as reported in **Table 3**. Sex analysis revealed a significant difference in childhood clinical care (Pearson chi2 = 6.23, p = 0.013), more frequently reported by males, and in previous pharmacological treatment (Pearson chi2 = 12.34, p = 0.015). No significant gender differences emerged regarding psychiatric or medical comorbidities, substance misuse, or psychiatric and non-psychiatric hospitalizations.

At baseline (T0), 40.35% of patients were not receiving pharmacological treatment. Among those under treatment, antipsychotics were the most used class (42.11%), followed by antidepressants (15.79%) (**Table 2**). Among the patients treated with methylphenidate, most of them did not receive any pharmacological treatment (69.70%, RS ≥2, p<0.05), followed by patients receiving antidepressants (27.27%, RS ≥2, p<0.05), whereas among the patients treated with atomoxetine, 24 of them (82.76%, RS ≥2, p<0.05) received antipsychotics, in a statistically significant difference (Pearson chi2 = 57.00, p< 0.001). At T0, psychiatric comorbidity was present in 63.16% of the sample, most frequently mood and anxiety disorders (33.3%), followed by personality disorders (17.54%), bipolar disorders (5.26%) and autism spectrum disorder (5.26%). No statistically significant differences were highlighted between the two treatment groups (**Table 2**). Organic comorbidities (5.26%%) and substance use (8.77%) were less frequent conditions, with no statistically significant differences highlighted between the two treatment groups (**Table 2**).

Most patients (59.56%) were followed by an outpatient health service at the time, mainly Community Mental Health Centers (36.8%) or private specialists (24.6%), with a statistically significant difference between the two treatment groups (72.41% of atomoxetine group vs 46.43% metylphenidate group, Pearson chi2 = 3.10, p = 0.046). Only 15.79% of patients had been previously hospitalized in a psychiatric ward, with no statistically significant differences highlighted between the two treatment groups (**Table 2**).

### Comparison between methylphenidate and atomoxetine treatments

In **Table 4**, the starting and final titration dose of methylphenidate and atomoxetine have been reported. The titration time did not statistically significantly differ between the two treatment groups, whereas discontinuation treatment was reported only among patients treated with atomoxetine, due to adverse effect (21.43%), followed by inefficacy (10.71%) and by both these conditions (7.14%) (**Table 4**).

**Table 4:** Pharmacological variables of the sample divided into the two treatment groups.

	Methylphenidate (n= 28)	Atomoxetine (n=29)	Statistical test	Probability
Starting dose (mg), m ± SD	18.39 ± 12.55	40.21 ± 9.76		
Final Titration dose (mg), m ± SD	25.89 ± 11.14	75.34 ± 17.32		
Titration period (days), m ± SD	151.71 ± 153.14	195.21± 334.63	*t* = -0.63, *t*-test	p = 0.5332
Drop out				
Present	0 (0%)	10 (35.71%)	Pearson chi2 = 11.71	p = 0.001
Not present	28 (100%)	19 (65.52%)		
Drop out reasons				
Adverse effects	0 (0%)	6 (21.43%)		
Inefficacy	0 (0%)	3 (10.71%)		
Adverse effects + inefficacy	0 (0%)	2 (7.14%)		
Period of adverse effects comparison				
Days of treatment	0 (0%)	206.17 ± 181.25		
Period of treatment				
Days, m ± SD	143.28 ± 81.31	208.31± 227.83	*t* = -1.42, *t*-test	p = 0.1599
Period of treatment divided by the median (136 days)				
≤ median	14 (50%)	14 (48%)	Pearson Chi2 = 0.02	p = 0.896
> median	14 (50%)	15 (52%)		

The mean periods of the two treatments did not statistically significantly differ (*t* = -1.42, *t*-test, p = 0.1599), nor did the periods of the two treatments divided according to the median (136 days) (Pearson Chi2 = 0.02; p = 0.896).

At follow-up (T1), methylphenidate treatment was associated with significantly higher AAQoL-29 total scores and higher scores in the Productivity, Psychological Health, and Life Outlook subscales compared with atomoxetine (**Table 5**). Similarly, RSES total score and Personal Satisfaction score were significantly higher in the methylphenidate group (**Table 5**). ADHD symptomatology was significantly reduced in the methylphenidate group compared with the atomoxetine group, as highlighted by the BAARS Quick Screen score reduction at T1.

**Table 5:** Comparison of psychometric scale scores between the two treatment groups at T1.

Scale	Methylphenidate (mean ± SD)	Atomoxetine (mean ± SD)	t-student	Probability
	T1	T1	*t*	p-value
AAQoL total score	1644.6 ± 364.92	1384.5 ± 322.55	2.85	0.006
Productivity (AAQoL)	601.8 ± 161.29	512.1 ± 157.92	2.12	0.038
Psychological Health (AAQoL)	325.9 ± 104.85	269.8 ± 103.16	2.03	0.047
Interpersonal Relationships (AAQoL)	325.9 ± 92.67	279.3 ± 98.67	1.84	0.072
Life Outlook (AAQoL)	391.1 ± 119.84	326.7 ± 101.54	2.19	0.033
Rosenberg Self-Esteem (RSES) total score	18.3 ± 5.01	15.1 ± 5.67	2.22	0.031
Personal Competence	10.0 ± 2.57	8.6 ± 3.06	1.93	0.059
Personal Satisfaction	8.32 ± 2.87	6.55 ± 2.77	2.37	0.021
GAF	67.6 ± 5.37	68.8 ± 6.04	0.83	0.410
BAARS Quick Screen	11,6 ± 3.18	13.8 ± 3.19	-2.63	0.011

### Psychometric scales at baseline (T0) and at follow-up (T1): difference between the two sexes

At baseline (T0), no significant sex differences emerged in DIVA-5 scores for inattention and hyperactivity/impulsivity in either adulthood or childhood. Similarly, BAARS total scores and childhood symptom scores showed no significant sex differences. Quality of life analysis revealed significant differences: men reported higher AAQoL-29 total scores (M = 1253.8 ± 495.4) than women (M = 997.6 ± 382.2; t = 2.20; p = 0.032), as well as higher scores in the Productivity and Daily Life (t = 2.49; p = 0.016) and Relationships (t = 2.19; p = 0.033) subscales. No significant differences emerged for Psychological Health or Life Outlook (**Table 6**). No significant gender differences were observed in baseline self-esteem (RSES total scores).

**Table 6:** Psychometric scales (AAQoL and RSES) at T0 and T1 in males and females of our sample.

	Males (m ± SD)	Females (m ± SD)	*t* (*t*-student)	Probability
T0				
AAQoL total score	1253.85 ± 495.41	997.58 ± 382.17	2.20	0.032
Productivity and Daily Life (AAQoL)	466.35 ± 218.17	336.29 ± 176.29	2.49	0.016
Psychological Health (AAQoL)	240.38 ± 137.85	179.84 ± 117.87	1.79	0.079
Interpersonal Relationships(AAQoL)	271.15 ± 107.63	212.16 ± 95.94	2.19	0.033
Life Outlook (AAQoL)	275.96 ± 131.81	260.48 ± 129.55	0.45	0.658
Rosenberg Self-Esteem (RSES)	15.15 ± 5.53	12.69 ± 5.44	1.69	0.095
Personal Competence	8.53 ± 2.64	7.54 ± 2.67	1.40	0.167
Personal Satisfaction	6.65 ± 3.29	5.13 ± 2.99	1.83	0.072
T1				
AAQoL total score	1584.62 ± 359.03	1451.61 ± 365.14	1.38	0.173
Productivity and Daily Life (AAQoL)	586.54 ± 173.67	530.65 ± 154.61	1.29	0.204
Psychological Health (AAQoL)	310.58 ± 112.07	286.29 ± 102.84	0.85	0.397
Interpersonal Relationships (AAQoL)	334.62 ± 77.48	275 ± 105.67	2.39	0.020
Life Outlook (AAQoL)	352.88 ± 96.52	362.90 ± 129.24	- 0.32	0.746
Rosenberg Self-Esteem (RSES)	18.73 ± 5.85	14.97 ± 4.70	2.69	0.009
Personal Competence	10.50 ± 3.02	8.29 ± 2.41	3.07	0.003
Personal Satisfaction	8.31 ± 3.12	6.68 ± 2.59	2.16	0.035

At follow-up (T1), some gender differences observed at baseline persisted or became more pronounced, as reported in **Table 6**. In particular, the AAQoL-29 subscale Relationships remained significantly higher in men (t = 2.39; p = 0.020) compared with women (275 ± 105.67). Self-esteem measured by the RSES showed significantly higher mean scores in males, as well as higher scores in the Personal Competence and Self-Satisfaction subscales, indicating a more positive self-perception among men. Overall, these findings suggest that, despite clinical improvement, women with ADHD continue to report lower levels of self-esteem and quality of life compared with men (**Table 6**).

### Longitudinal changes between T0 and T1

Longitudinal analysis revealed statistically significant improvements between T0 and T1 across all evaluated dimensions (**Table 7**). Overall quality of life (AAQoL-29) showed a significant increase in total score (from 1114.47 ± 452.1 to 1512.28 ± 365.29), accompanied by significant improvements in all subscales: Productivity and Daily Life, Psychological Health, Relationships, and Life Outlook.

**Table 7:** Comparison of psychometric scale scores between T0 and T1.

Psychometric Scales	T0	T1	*t*-student	Probability	
	Mean ± SD	Mean ± SD	*t*	p-value	
Full BAARS-IV and BAARS-IV Quick Screen					
Current Symtoms	14.14 ± 4.29	12.74 ± 3.35	2.40	0.0199	
AAQoL-29					
Total	1114.47 ± 452.10	1512.28 ± 365.29	-5.76	<0.001
Productivity of Daily Life	395.61 ± 205.35	556.14 ± 164.49	-5.31	<0.001
Psychological Health	207.46 ± 129.81	297.37 ± 106.87	-4.67	<0.001
Interpersonal Relationships	239.07 ± 104.79	302.19 ± 97.77	-3.81	0.0003
Life Outlook	267.54 ± 129.64	358.33 ± 114.60	-4.81	<0.001
RSES					
Total	13.81 ± 5.58	16.68 ± 5.54	-4.05	0.0002
Personal Competence	8.00 ± 2.68	9.30 ± 2.90	-3.46	<0.001
Personal Satisfaction	5.82 ± 3.19	7.42 ± 2.93	-4.05	0.0002
GAF					
	60.70 ± 5.69	68.21 ± 5.71	11.70	<0.001

Global functioning (GAF) also improved significantly, with a substantial mean score increase. Self-esteem demonstrated significant improvement over time, both in total RSES scores and in the Personal Competence and Self-Satisfaction subscales. Conversely, no significant changes were observed in the self-criticism dimension.

Subjective ADHD symptomatology assessed through Current Symptoms in the full BAARS IV Self-Report at T0 and Current Symptoms in BAARS IV Quick Screen at T1 showed a significant reduction, indicating a perceived clinical improvement associated with pharmacological treatment (**Table 7**).

### Correlation analyses

Correlation analyses showed that greater attentional impairment assessed by DIVA-5 was associated with greater subjective symptom severity and lower levels of quality of life and self-esteem (**Table 8**). Total ADHD symptom severity was inversely correlated with self-esteem and multiple quality of life domains whereas quality of life and self-esteem measures showed to be positively related to each other.

**Table 8:** Correlations between psychometric scales (Spearman rho).

Spearman’s rho probabilitày	DIVA 5 inattention adulthood	DIVA 5 inattention childhood	DIVA 5 hyperactivity/impulsivity adulthood	DIVA 5 hyperactivity/impulsivity childhood	Full BAARS IV current symptoms	Full BAARS IV childhood symptoms	Full BAARS IV total score	RSES total score	AAQoL29 total score
DIVA 5 Inattention Adulthood	–	–	–	–	0.346 p=0.0084	Not Significant	0.274 p=0.0394	-0.327 p=0.0131	-0.376 p=0.0040
DiVA 5 Inattention Childhood	–	–	–	–	Not Significant	0.452 p=0.0004	Not Significant	Not Significant	Not Significant
DIVA 5 Hyperactivity/Impulsivity Adulthood	–	–	–	–	Not Significant	Not Significant	Not Significant	Not Significant	Not Significant
DIVA 5 Hyperactivity/Impulsivity Childhood	–	–	–	–	-0.304 p=0.0216	Not Significant	Not Significant	Not Significant	Not Significant
Full BAARS IV Current Symptoms	0.346 p=0.0084	Not Significant	Not Significant	-0.304 p=0.0216	–	Not Significant	0.782 p<0.0001	Not Significant	Not Significant
Full BAARS IV Childhood Symptoms	–	0.452 p=0.0004	Not Significant	Not Significant	Not Significant	–	0.652 p<0.0001	Not Significant	Not Significant
Full BAARS IV Total Score	0.274 p=0.0394	Not Significant	Not Significant	Not Significant	0.782 p<0.0001	0.652 p<0.0001	–	0.327 p=0.0132	Not Significant
RSES total score	-0.327 p=0.0131	Not Significant	Not Significant	Not Significant	Not Significant	Not Significant	-0.327 p=0.0132	–	0.636 p<0.0001
AAQoL29 total score	-0.376 p=0.0040	Not Significant	Not Significant	Not Significant	Not Significant	Not Significant	Not Significant	0.636 p<0.0001	–

## Discussion

The present study suggests the central role of pharmacological therapy in the treatment of adult ADHD, highlighting significant improvements not only in core symptomatology but also in other dimensions such as quality of life, self-esteem, and global functioning. The findings suggest that pharmacological treatment, regardless of the active compound used, can produce benefits extending beyond symptom control, substantially influencing self-perception, personal efficacy, and psychosocial adjustment.

These results support a conceptualization of adult ADHD as a complex neuropsychological condition involving not only attention and impulsivity, but also emotional regulation, personal identity, and self-efficacy. Within this framework, symptom reduction represents a necessary but insufficient condition for patient well-being; clinically meaningful improvement involves the restoration of a sense of internal coherence and competence, elements that define quality of life and psychological stability in adults with ADHD.

### Sample characteristics and clinical implications

Analysis of the socio-demographic and clinical characteristics highlights the predominance of young adults, with a mean age below 40 years, confirming that ADHD continues to exert a significant impact beyond developmental age, interfering with identity consolidation, occupational integration, and the establishment of stable intimate relationships. This finding is consistent with epidemiological evidence describing ADHD as a lifespan condition (42).

The greater male representation aligns with existing literature but simultaneously suggests possible underdiagnosis in females, in whom the disorder often presents more internalizing profile characterized by inattention, anxiety, and low self-esteem rather than overt hyperactivity (43, 44). This observation underscores the need for greater clinical sensitivity in identifying ADHD in women, where the condition is often associated with quieter yet chronic suffering and a substantial impact on quality of life.

The relatively high educational level of the sample, coupled with frequent occupational instability or professional dissatisfaction, highlights one of the typical paradoxes of adult ADHD: the discrepancy between cognitive potential and actual performance. Difficulties in planning, procrastination, and emotional self-regulation hinder the translation of abilities into concrete outcomes, fostering long-term frustration and self-devaluation. This dynamic appears particularly relevant to self-esteem development, as supported by the observed correlations between ADHD symptomatology and psychological well-being.

The frequent presence of anxiety–depressive comorbidity and moderate impairment in global functioning confirms the multidimensional nature of adult ADHD. These conditions not only exacerbate the clinical picture but directly affect subjective well-being, underscoring the need for therapeutic approaches that integrate treatment of cognitive symptoms with emotional and relational dimensions.

The two treatment groups showed similar demographic and clinical variables, representing two comparable homogeneous groups.

### Effects of pharmacological therapy on self-esteem and quality of life

One of the most relevant findings of the study is the significant improvement in Rosenberg Self-Esteem Scale (RSES) and Adult ADHD Quality of Life Scale (AAQoL-29) scores, accompanied by increased global functioning (GAF).

These data indicate that pharmacological therapy exerts a positive effect on self-perception and the capacity to manage daily life demands.

Correlation analyses showed that ADHD symptom severity, particularly attentional deficits, is inversely associated with self-esteem, perceived competence, personal satisfaction, and psychological health. These findings suggest that symptom reduction achieved through pharmacological treatment directly contributes to improvements in self-concept and perceived personal efficacy.

This relationship can be interpreted in light of Bandura’s self-efficacy theory (1977), which posits that perceived control over one’s actions constitutes a fundamental basis for self-esteem development (45). In ADHD, chronic difficulties in sustaining attention, meeting obligations, and regulating emotions foster the development of learned helplessness, undermining confidence in one’s abilities. A recent study has highlighted that baseline ADHD symptoms predicted more frequent Average Automatic Thought, and more severe task avoidance and negative emotion in daily life (46). A positive response to pharmacological therapy, particularly with methylphenidate, appears to interrupt this vicious cycle, enabling individuals to experience success and progressively restructure their self-image. From a cognitive-behavioral perspective, this process may be conceptualized as “experiential relearning” (47), whereby dysfunctional beliefs are modified through concrete evidence of improved functioning. From a psychodynamic perspective, reductions in impulsivity and improvements in attentional control may act as corrective emotional experiences, promoting the reworking of narcissistic injuries and greater self-cohesion (48, 49). From a neurobiological standpoint, these changes can be explained by dopaminergic and noradrenergic modulation of the prefrontal cortex and fronto-limbic networks involved in attention, planning, and emotional regulation (50, 51). Improved executive functioning translates into enhanced self-regulation, increased productivity, and improved interpersonal relationships, as observed in previous studies (16, 52).

### Differences between methylphenidate and atomoxetine

The two treatment groups were comparable in terms of sample size and most demographic and clinical characteristics, with a few exceptions. In particular, the methylphenidate group included a higher proportion of males, a greater percentage of self-employed individuals, and fewer patients receiving concomitant psychopharmacological medications, although antidepressant use was more frequent. Conversely, the atomoxetine group included a higher proportion of females and was more frequently treated with antipsychotic medications. Among children and adolescents, the two treatment groups differed regarding age at first clinical evaluation, which was younger in the atomoxetine group. In addition, patients in the atomoxetine group more frequently received educational and rehabilitative interventions, suggesting an earlier onset and a more severe clinical presentation of ADHD.

The comparison between methylphenidate and atomoxetine indicates that both medications produce significant clinical improvement through different neurobiological mechanisms. Methylphenidate, a dopamine and norepinephrine reuptake inhibitor, primarily acts on fronto-striatal circuits and the anterior cingulate cortex, resulting in rapid improvements in cognitive performance, sustained attention, and inhibitory control. In the present study, this translated into greater reductions in ADHD symptom severity (lower BAARS-IV scores) and higher quality of life and self-esteem scores. Atomoxetine, a selective norepinephrine reuptake inhibitor, acts predominantly on the dorsolateral prefrontal cortex and its connections with the limbic system, promoting more gradual and stable emotional regulation with improved quality of life (53–55). Subjects treated with atomoxetine showed higher levels of self-esteem, suggesting greater tolerance toward personal limitations and reduced self-criticism. These differences may be interpreted in light of Siegel’s neuro-integration theory (2012) (56), according to which psychological well-being arises from the balance between higher cortical functions and limbic emotional processes. Methylphenidate primarily enhances attentional and motivational networks, whereas atomoxetine promotes integration between cognitive and emotional regulation. Clinically, this suggests that methylphenidate may be preferable in patients with marked cognitive deficits, whereas atomoxetine may be particularly indicated in the presence of anxiety–depressive comorbidity or emotional dysregulation.

### Sex differences and psychological implications

The observed gender differences indicate greater emotional vulnerability in women with ADHD, who reported higher current symptom levels and lower quality of life and self-esteem scores. These findings are consistent with literature describing female ADHD as a more internalizing phenotype, often associated with feelings of inadequacy and failure (43, 44). Clinically, these findings highlight the need for gender-sensitive therapeutic approaches integrating pharmacotherapy with psychotherapeutic interventions aimed at strengthening self-esteem and emotional regulation. Mindfulness-based interventions have shown promising results as complementary interventions (57). Overall, the correlations observed between ADHD symptomatology, self-esteem, and quality of life support the need for an integrated therapeutic model in which pharmacotherapy provides the neurochemical foundation upon which psychotherapeutic interventions targeting self-restructuring and self-efficacy enhancement can be built. The literature supports the effectiveness of combined approaches integrating pharmacotherapy with ADHD-specific cognitive-behavioral therapy (9, 58, 59), as well as mindfulness-based and dialectical-behavioral interventions (51, 60).

### Study limitations and advantages

This study has several limitations, particularly the small sample size, limited follow-up duration, and the naturalistic and single-center design. In particular, T1 is a non-homogeneous time-point due to the naturalistic design of the study and the need to collect as many patients as possible. It represents one of the main limitations of this study. The lack of a placebo control group prevents us from completely ruling out the placebo effect, which is present in all treatments and could explain the improvement of psychological dimensions in our sample. However, one advantage of the study could be the direct comparison of two pharmacological treatments between two comparable groups in a real-life setting, along with its focus on the well-being of people with ADHD in adulthood.

## Conclusions

In conclusion, the findings of the present study suggest that pharmacological therapy in adult ADHD not only reduces symptoms but also promotes significant improvements in quality of life and self-esteem, key components of psychological well-being and social integration. Our study highlights that the severity of ADHD symptomatology in adulthood, particularly within the attentional domain, is inversely associated with quality of life, self-esteem, and personal satisfaction, whereas symptom reduction is accompanied by a significant improvement in subjective well-being. Pharmacological therapy can be considered as a fundamental pillar in the treatment of adult ADHD, demonstrating a positive impact not only on core symptoms but also on central psychological dimensions such as perceived personal efficacy and self-image. Both medications examined showed benefits in global functioning and subjective well-being, albeit through different mechanisms of action: methylphenidate proved more effective in improving cognitive efficiency and productivity, whereas atomoxetine demonstrated a more gradual effect on emotional regulation and self-competence although with more frequent adverse effects and drop-out. The findings of this study also highlight greater emotional vulnerability among women with ADHD, underscoring the need for gender-sensitive therapeutic approaches integrated with targeted psychological interventions.

In conclusion, the treatment of adult ADHD should be conceptualized as an integrated pathway aimed not only at symptom reduction but also at restoring self-esteem, improving quality of life, and promoting full integration of the self.

## Data Availability

The authors selected the following statement: The raw data supporting the conclusions of this article will be made available by the authors, without undue reservation.
